# Screening for genetic variability in photosynthetic regulation provides insights into salt performance traits in forage sorghum under salt stress

**DOI:** 10.1186/s12870-024-05406-9

**Published:** 2024-07-19

**Authors:** Erick Amombo, Maryam Gbibar, Dennis S. Ashilenje, Abdelaziz Hirich, Lamfeddal Kouisni, Abdallah Oukarroum, Cherki Ghoulam, Mohamed El Gharous, Abdelaziz Nilahyane

**Affiliations:** 1 African Sustainable Agriculture Research Institute (ASARI), Mohammed VI Polytechnic University (UM6P), Laâyoune, Morocco; 2 AgroBioSciences Department (AgBS), Mohammed VI Polytechnic University (UM6P), Ben Guerir, Morocco; 3 Center of Agrobiotechnology and Bioengineering, Labeled Research Unit CNRST, Cadi Ayyad University (UCA), Marrakech, Morocco; 4 Agricultural Innovation and Technology Transfer Center (AITTC), Mohammed VI Polytechnic University (UM6P), Ben Guerir, Morocco

**Keywords:** Genetic diversity, Growth stage, Photosynthetic performance index, *SbrbcL8*, Yield, Salt performance index

## Abstract

**Background:**

Sorghum (*Sorghum bicolor*) is a promising opportunity crop for arid regions of Africa due to its high tolerance to drought and heat stresses. Screening for genetic variability in photosynthetic regulation under salt stress can help to identify target trait combinations essential for sorghum genetic improvement. The primary objective of this study was to identify reliable indicators of photosynthetic performance under salt stress for forage yield within a panel of 18 sorghum varieties from stage 1 (leaf 3) to stage 7 (late flowering to early silage maturity). We dissected the genetic diversity and variability in five stress-sensitive photosynthetic parameters: nonphotochemical chlorophyll fluorescence quenching (NPQ), the electron transport rate (ETR), the maximum potential quantum efficiency of photosystem II (F_V_/F_M_), the CO_2_ assimilation rate (A), and the photosynthetic performance based on absorption (PI_ABS_). Further, we investigated potential genes for target phenotypes using a combined approach of bioinformatics, transcriptional analysis, and homologous overexpression.

**Results:**

The panel revealed polymorphism, two admixed subpopulations, and significant molecular variability between and within population. During the investigated development stages, the PI_ABS_ varied dramatically and consistently amongst varieties. Under higher saline conditions, PI_ABS_ also showed a significant positive connection with A and dry matter gain. Because PI_ABS_ is a measure of plants’ overall photosynthetic performance, it was applied to predict the salinity performance index (SPI). The SPI correlated positively with dry matter gain, demonstrating that PI_ABS_ could be used as a reliable salt stress performance marker for forage sorghum. Eight rubisco large subunit genes were identified in-silico and validated using qPCR with variable expression across the varieties under saline conditions. Overexpression of *Rubisco Large Subunit 8* increased PI_ABS_, altered the OJIP, and growth with an insignificant effect on A.

**Conclusions:**

These findings provide insights into strategies for enhancing the photosynthetic performance of sorghum under saline conditions for improved photosynthetic performance and potential dry matter yield. The integration of molecular approaches, guided by the identified genetic variability, holds promise for genetically breeding sorghum tailored to thrive in arid and saline environments, contributing to sustainable agricultural practices.

**Supplementary Information:**

The online version contains supplementary material available at 10.1186/s12870-024-05406-9.

## Introduction

Sorghum (*Sorghum bicolor* L. Moench) is a globally cultivated and consumed major cereal crop [1]. Its increasing preference among both small- and large-scale farmers is attributed to its potential for higher and more consistent yields on marginal lands than other cereals [2]. In addition, the versatility of sorghum as a forage, feed, food, and industrial crop has made it to rapidly gain popularity in Africa as an alternative crop [3]. However, despite its high potential, sorghum acreage still lags that of other cereals [4]. This discrepancy is attributed to poor variety selection [5] and the sluggish introduction of improved varieties capable of adapting to rapidly changing environments [6]. Thus, there is an urgent need to avail phenotypic and genomic resources to introduce new and improved varieties tailored to these challenging environments.

The challenges faced by agriculture in Africa extend beyond meeting the demands for food and feed to proper land management for long-term sustainability [7]. Increasingly, rising soil and water salinity because of poor irrigation practices constitutes a significant abiotic factor limiting crop performance and yields [8]. By 2024, salinity affects approximately 25% of Africa’s geographical surface area, and this proportion is expanding from natural and anthropogenic causes [9]. The long-term ramifications are substantial, especially if the minimal arable portion cannot be adequately managed to sustain more than twice its existing population [10]. Furthermore, the uneven distribution of salinity complicates the design of regional-level mitigation policies [11]. Compared to those of other crops, genetic studies of sorghum have yet to yield meaningful genetic improvement outcomes in the context of forage yield and tolerance to salt stress [12]. Nonetheless, the positive aspect of this challenge lies in the extensive intraspecies diversity in sorghum performance under diverse abiotic stresses, which offers a rich genetic resource for realizing these genetic improvement goals [13].

Photosynthesis is overly sensitive to salt stress [14]. As the primary centers for photosynthesis, the leaves constitute 90% of the total plant chlorophyll, which comprises the primary light-harvesting complex responsible for initiating photochemical events [15]. Salinity induces physiological drought, leading to a disruption in the equilibrium between leaf transpiration and root water uptake [16]. Salt-stressed leaves undergo reduction‒oxidation of photosystem II acceptors, consequently diminishing the photosynthetic electron transport efficiency in both PSI and PSII [17]. In sorghum, salinity induces a reduction in the photochemical efficiency of PSII and stomatal conductance, direct interference with the photosynthetic apparatus, and the inhibition of overall plant photosynthesis [18]. Sui et al. [19] observed a disruption in the light-harvesting complex, early CO_2_ fixation in mesophyll cells, and NADP-malate dehydrogenase-mediated oxidative decarboxylation of malic acid to transfer CO_2_ to Rubisco. A holistic analysis of these reports suggested that photosynthesis under salt stress is governed by genes that function complementarily or conversely, particularly in a delicate balance involving light harvesting, photochemical reactions, CO_2_ assimilation, and net growth, thereby influencing overall growth and yield. Hence, photosynthesis can serve not only as a significant stress biomarker for sorghum but also as a basis for engineering salt-tolerant and high-yielding forage sorghum varieties.

A comprehensive examination of the behavior of salt-responsive phenotypes across various growth stages preceding silage harvesting is the initial step in discerning reliable phenotypic and molecular predictors of salt tolerance and forage improvement. Sorghum plants exhibit substantial genetic variation in salt response-related properties, including ion homeostasis [20], germination vigor [21], agronomic traits [22], and metabolite changes [23]. In this study, an analysis of the genetic variation in stress-sensitive photosynthetic characteristics was conducted on sorghum varieties with the aim of identifying reliable photosynthetic indicators of salt stress performance for forage yield.

## Materials and methods

The plant materials were composed of 18 varieties comprising 13 local germplasms sourced from the National Institute for Agricultural Research (INRA), Morocco, as well as 2 inbred lines and 3 commercial varieties (Additional File 1). The commercial accessions are forage hybrids popular with local farmers. The two inbred lines are drought tolerant accessions from the International Crops Research Institute for the Semi-Arid Tropics (ICRISAT) which we acquired for salinity trials. The local accessions are drought-tolerant landraces provided by the local gene bank for salinity trials. Trials were conducted at two distinct platforms: a low-salinity platform with naturally saline irrigation water with electrical conductivity of 4 dS/m (low salinity) and 8 dS/m (medium salinity platform). The experimental design employed a randomized complete block design, with plots measuring 6 × 2 m and a seeding rate of 20 seeds/m^2^. Drip irrigation was applied to the plants every other day, maintaining optimal moisture conditions throughout the duration of the experiment. Planting took place during the spring seasons of 2021 and 2022 in Laayoune region, Morocco. The study area is characterized by a dry climate with less than 30 mm of rainfall annually, and the temperature normally ranged from 14 °C to 29 °C.

### Selection of growth stages

Three distinct growth stages were chosen for evaluation: leaf 3, boot, and flowering. The leaf 3 stage signifies a critical stage in sorghum growth when the leaves are expanding to maximize light interception [24]. Notably, variations in growth, greenness, and overall shoot health became apparent at this stage.

At the boot stage, the leaves fully expand, and photosynthetic light interception is optimized. The head is full-sized and enclosed within the flag leaf sheath. This stage is particularly crucial for determining reproductive success, as salt-sensitive varieties tend to exhibit slower heading. Final measurements were conducted at the full flowering stage, representing the last physiological stage before the soft dough stage recommended for silage harvest according to Gerik et al. [25]. Morphologically, at this stage, the peduncles of all the varieties elongate, pushing the head through the flag leaf sheath. The effect of salinity on morphology was notable at this stage. These chosen growth stages and corresponding morphological assessments provide comprehensive insights into the impact of salinity on sorghum development and reproductive success and are crucial for understanding and optimizing crop performance under saline conditions.

### Photosynthesis measurement

Photosynthesis measurements were conducted using the LI-6800 Portable Photosynthesis System equipment (Li-COR Biosciences, Lincoln, NE 68504, United States). All the measurements were carried out in the morning after a 30-minute dark adaptation period. The assessments were performed on the attached ear leaves of six tasseling plants located in the middle of each plot. The ear leaf was selected due to its representation of the total canopy chlorophyll content. Chlorophyll fluorescence measurements were performed on both low- and medium-salinity platforms on the same day during the morning hours to minimize time wastage. Following the adaptation of the leaves to darkness for 30 min, a light pulse at a flow rate of 550 µmol/m^2^/s was applied using a light-emitting diode. The fast fluorescence kinetics (F_0_ to F_M_) were recorded from 10 µs to 1 s. Six repetitions were applied for each variety and treatment to ensure robust data collection. The acquired data were analyzed using the JIP test according to the methodology outlined by Force et al. [26], as:

$$\:FV/FM=\frac{FM-F0}{FM}$$

The performance index (PI_ABS_) was derived from the OJIP curve as follows:

$$\:PI\text{ABS}=\frac{1-\left(F\text{0}/F\text{M}\right)}{M\text{0}/Vj}\times\:\frac{\left(F\text{M}/F\text{0}\right)}{F\text{0}}\times\:\frac{1-\text{V}\text{j}}{Vj}$$

where F_0_ is the value of fluorescence at 50 ms, F_M_ is the value of fluorescence at its maximum, M_0_ is the value of the initial slope of fluorescence kinetics, calculated as follows: M_0_ = 4 (F300 s - F_0_)/(F_M_ - F_0_), V_J_ is the value of the relative variable fluorescence at 2 ms, calculated as follows: V_J_ = (F_J_ - F_0_)/(F_M_ - F_0_), and F_J_ is the fluorescence at 2 ms.

### Calculation of the salt performance index (SPI)

Assessing the magnitude of change in photosynthetic parameters across different varieties toward late physiological maturity, just before most sensitive varieties begin to decline under stress conditions, can provide insight into reliable physiological parameters for phenotyping a panel under a given stress. An ideal photosynthetic marker should be sensitive at both early and advanced growth stages to capture changes across all varieties before harvest [27]. To identify this marker in this study, we determined the reduction factor by calculating the ratio of photosynthetic parameters recorded at the leaf 3 to those recorded at the late flowering stage. In other words, there was a decrease in performance between early and late physiological activity before silage harvest. A more significant reduction factor across various assessed physiological traits in sorghum varieties clearly signifies heightened sensitivity to salt stress for each trait for the variety beyond which most sensitive varieties begin to succumb to stress; hence, this trait can be used to predict the performance index.

Therefore, the SPI operates under the premise that sorghum varieties with higher salt tolerance and capable to withstand salt stress until late flowering will maintain high selected photosynthetic performance values and accumulate more biomass than salt-sensitive varieties. Therefore, the SPI deems a reduction in the selected photosynthetic parameter during late flowering (LogB) to be more impactful, assigning *n* (reduction factor) the significance of a reduction at leaf 3 (LogA), hence expressed based on Oukarroum et al. [28] as follows:

$$\:SPI=LogA+nLogB$$

where A is the relative value of the photosynthetic parameter at leaf 3 (medium/low salinity), B is the relative value of the identified photosynthetic parameter (medium/low salinity) at late flowering, and *n* is the significant reduction factor (A/B).

### DNA extraction, PCR, and gel electrophoresis

The cetyltrimethylammonium bromide (CTAB) principle, as described by Doyle [29], was used for the extraction of whole-genome DNA from the leaves of 18 sorghum varieties. In brief, two-week-old sorghum leaves were collected, and 2.5 g of each plant sample was homogenized in 500 µL of a 5% CTAB buffer mixture. Subsequently, 2 µL of RNAse A and 5 µL of proteinase K were added to the samples, followed by heating to 56 °C for 30 min with intermittent gentle vortexing. After cooling to room temperature, equal quantities of phenol and chloroform (a total of 500 µL) were added to the samples, which were then gently vortexed for 1 min and inverted to ensure thorough mixing before centrifugation at 15,000 rpm for 20 min. The upper clear phase was carefully isolated and rewashed with equal volumes of phenol: chloroform solution, followed by another centrifugation at 15,000 rpm for 20 min. The pellet was precipitated by adding an ice-cold isopropanol: sodium acetate mixture at a ratio of 450:50 and then centrifuging at 14,000 rpm for 5 min. After removing the isopropanol, the pellet was desalted with 500 µL of 70% ethanol followed by absolute ethanol. The ethanol was vacuum evaporated, and the pellet was suspended in 70 µL of elution buffer. The DNA concentration and purity were checked with a NanoDrop spectrophotometer (VWR^®^ mySPEC, Microvolume Spectrophotometer).

For PCR and gel electrophoresis, 30 SSR markers distributed across the sorghum genome were used. The efficiency and specificity of the primers were evaluated via DNA club software. The SSRs were synthesized by Invitrogen (Thermo Fisher Scientific). PCR was carried out in a thermocycler (UNO96 VWR thermocycler) with a 20 µL reaction mixture using the PCR master mix 5x FIREPol^®^ Master Mix Ready To Load. The thermocycling procedure included 40 cycles of initial denaturation at 95 °C for 5 min, denaturation at 95 °C for 10 s, annealing for 30 s, and extension at 72 °C for 10 s. Gel electrophoresis was performed on a precast polyacrylamide gel (6%) using a VWR vertical electrophoresis system in 2 L of 1X TBE running buffer stained with SYBR Green 1. Electrophoresis was conducted at 100 V for two hours, and a 50 bp DNA ladder was used for estimating molecular weights. PCR products were visualized and analyzed using a G: Box Chemi XX9 UV transilluminator and GeneTools software.

### Genetic diversity, molecular variance, and transcriptional analysis

Each allele’s fragment length was scored based on its location relative to the 50 bp standard DNA ladder. Alleles were graded as present [1] or missing [0], and band sizes for each marker per genotype were evaluated as a/b, where ‘a’ is the top band and ‘b’ is the bottom band. Arlequin software was used to calculate the allele frequency, allele number per locus, gene diversity, observed heterozygosity, and polymorphism information content (PIC) for each SSR. Molecular variance analysis (AMOVA) using the Shannon statistics test [30] was also conducted with GenAlEx version 6.502 software [31]. The data from 30 SSR markers were entered into STRUCTURE 2.3.4 software [32] for population structure analysis. We used the Bayesian model-based clustering approach. To maintain Hardy–Weinberg and linkage equilibrium, individual genotypes were assigned to subpopulations. The STRUCTURE software was executed ten times, with a predefined number of population groups (k) ranging from 1 to 10. We employed admixture models with 10,000 MCMC (Markov Chain Monte Carlo) replications and a burn-in period of 10,000 for each run. The optimal population number was determined based on the maximum likelihood of the probability of data (LnP(D)) in the output, along with an ad hoc statistic (DK) derived from the second-order rate of change in LnP(D) between successive K values. Additionally, 15 independent runs were conducted, each comprising 100,000 iterations after a burn-in period of 100,000, with K values set from one to five.

The ribulose-1,5-bisphosphate carboxylase/oxygenase large subunit protein sequences of Arabidopsis (*Arabidopsis thaliana*) and rice were used as queries in a BLASTp sequence alignment against the sorghum genome retrieved from the database [33]. The presence of the conserved RNA recognition motif (RRM) domain in the N-terminus of the large subunit was verified using the SMART database [34]. A HMMER search [35] was used to extract family genes containing rubisco domains. Molecular characteristics such as molecular weight (MW), amino acid number (aa), isoelectric point (pI), and chromosomal position were also analyzed. The pre-built ngLOC model database (http://ngloc.unmc.edu/) was used to retrieve a web-based interface for predicting subcellular localization. Rubisco protein sequences in FASTA format were used to create predictions, and sorghum were used as the default. The MLCS (Multi-Localization Confidence Score) [36] was used to determine the prediction level of the top two sites.

For RNA extraction, cDNA synthesis, and RT‒qPCR, total RNA was extracted from young sorghum leaves, and cDNA was synthesized using the HiScript II One Step qRT‒PCR SYBR Green Kit. RT‒qPCR was performed using an AriaMx Real-Time PCR instrument. The relative expression levels of the rubisco family genes were calculated using the 2^−∆∆Ct^ method, with the elongation factor 1 (*EF1*) gene serving as a reference.

Sorghum transformation was performed via direct protoplast electroporation, using a modified protocol described by D’Halluin et al. [37]. Briefly, local variety 3 that displayed low photosynthetic performance and dry matter embryos were sterilized and treated with 0.3% macerozyme at pH 5.6 for 3 min. After treatment, the embryos were washed and transferred to a disposable cuvette containing 200 µL of phosphate-buffered saline. Within each cuvette, 15 µL of plasmid DNA, which was generated by incorporating *SbrbcL8* cDNA from commercial variety V18 with higher dry matter and robust photosynthetic indicators and the *β-glucuronidase* reporter gene, was introduced into the enzyme-treated embryos. Following a 1-hour incubation period, the cuvettes were subjected to ice bath treatment for 10 min, followed by electroporation featuring a single pulse discharge with a field strength of 375 V/cm, facilitated by a 900-pF capacitor (BTX Twin Waveform Electroporation Systems, Holliston, MA, USA). After electroporation, the embryos were thoroughly washed and reintroduced into nutrient media to facilitate continued growth. Upon germination, the plants were transplanted into pots containing commercial soil and irrigated at regular intervals with a predetermined quantity of water. Experimental treatments were established, with both the overexpressed and wild-type plants exposed to saline water at a salinity level of 8 dS/m, while the control group received deionized water (EC = 0 dS/m). Any extra saline water was allowed to drain beneath the surface, with subsequent reirrigation until no further water was removed. Regular EC measurements were recorded to ensure a sustained and consistent salinity level.

The environmental conditions within the growth chamber were optimized to include a 14-hour photoperiod, a dark/light temperature cycle maintained at 25/30°C, and a relative humidity range of 55–65%. To simulate field conditions, the plants were irrigated with water of varying salinity levels, specifically 0 dS/m (deionized water) and 8 dS/m (NaCl). The 0 dS/m treatment serves as a control to observe the baseline growth and health of the plants without any salt stress while the 8 dS/m treatment was used to simulate salinity conditions and test the plants’ tolerance and response to salt stress. Phenotypic distinctions manifested in the plants after 8 days of treatment coincided with the results of the physiological analyses. To validate the efficacy of electroporation, fresh leaf samples were procured from the pots, quartered, and subsequently immersed in 0.5 mL of 5-bromo-4-chloro-3-indolyl-beta-D-glucuronic acid, cyclohexylammonium salt stain. These sections were then subjected to an overnight incubation at 37 °C, followed by rinsing in warm 70% ethanol until the chlorophyll color dissipated.

### Statistical analyses

The analysis utilized a one-way ANOVA model with six biological replicates to assess the differences among group means of the phenotypic variables. Each variable was treated as a dependent variable, and the variety was specified as the independent variable. The significance level was set at *p* ≤ 0.01. Significant differences identified through ANOVA were subjected to Duncan test to determine specific group differences. The analysis and graphing were performed with appropriate packages in R (version 4.3.2) with the *stats* [38], and *agricolae* [39] packages used for ANOVA and Duncan post-hoc test, respectively. The OJIP curves were generated using Origin Lab Pro (2022 release).

## Results

### Genetic diversity, population structure and molecular variance

The number of alleles per locus ranged from 2 to 5 with a total of 109 alleles successfully amplified from 30 polymorphic markers, resulting in an average of 3.63 alleles per locus. The observed heterozygosity displayed a broad range, varying from 0.18 to 0.41 across the accessions. The polymorphism information content (PIC) values ranged from 0.3 to 0.6, with a mean of 0.41, indicating a high level of polymorphism within the accessions (Additional File 2).

**Fig. 1:**
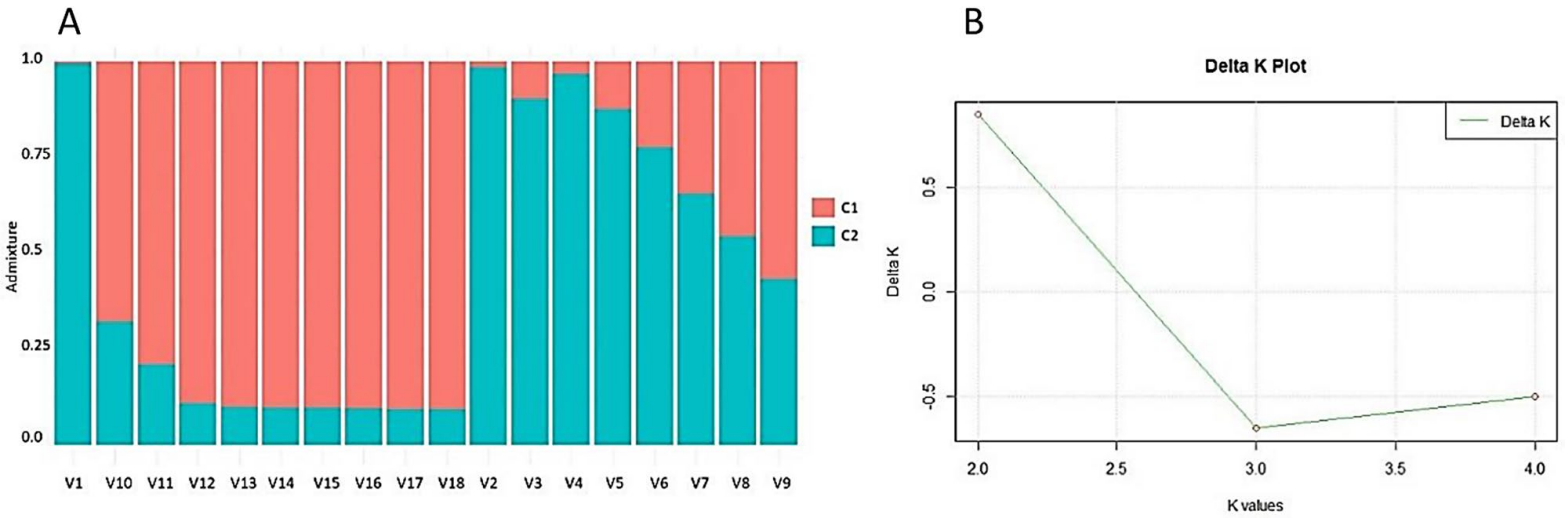
(A) Population structure matrix of sorghum varieties. Each vertical bar represents an individual, while different colors represent genetic clusters inferred by STRUCTURE analysis. Individuals are grouped based on their genetic similarity. The y-axis denotes the estimated proportion of genetic ancestry from each identified cluster. (B) The delta K plot indicates the most likely number of distinct populations in the dataset

Population structure analysis conducted using STRUCTURE identified two distinct subpopulations with considerable admixture. The results aligned with the preliminary run outcomes, showing an increase in the average probabilities of the data likelihoods for the population structure in the panel of accessions with increasing K values. Therefore, the probable number of subpopulations could also be identified utilizing the delta K method. Most local varieties were assigned to group 2, while 10, 11, 12 and 13 displayed higher admixtures levels in group 1. In contrast, all the hybrids and inbred lines clustered within group 1 (Fig. 1). These findings suggested potential common genomic regions shared among the accessions, especially within each respective group.

The results of the chi-square G test for variation among populations reveal insights into the distribution of variance in the sorghum population. Two distinct sources of variation were considered: “among population” and “within population,” with degrees of freedom of 2 and 14, respectively. The chi-square G test statistics for these categories were 33.72 and 19.82, respectively, totaling 53.54 for the entire dataset. Variance components indicated that “among population” variance surpassed that of the “within population” variance, resulting in a total variance of 5.43. Importantly, the variation proportion analysis illustrated that a substantial 71.7% of the total variance raised from differences among populations, while the remaining 28.3% accounts for within-population variation. Both components demonstrate statistical significance at *P* < 0.001 (Table 1).

**Table 1:** Shannon statistics of molecular variance partitioned by population loci

Source of variation	Degree of freedom	Chi-square G test	Variance components	Variation proportion (%)	^β^*P* value
Among pop.	2	33.72	3.84*	71.7	< 0.001
Within pop.	14	19.82	1.59*	28.3	< 0.001
**Total**	**16**	**53.54**	**5.43**		

^β^1000 permutations were used to determine P values under the null hypothesis that a specified variance component is zero

### Variation in the slow kinetics of chlorophyll a fluorescence and CO_2_ assimilation

**Fig. 2:**
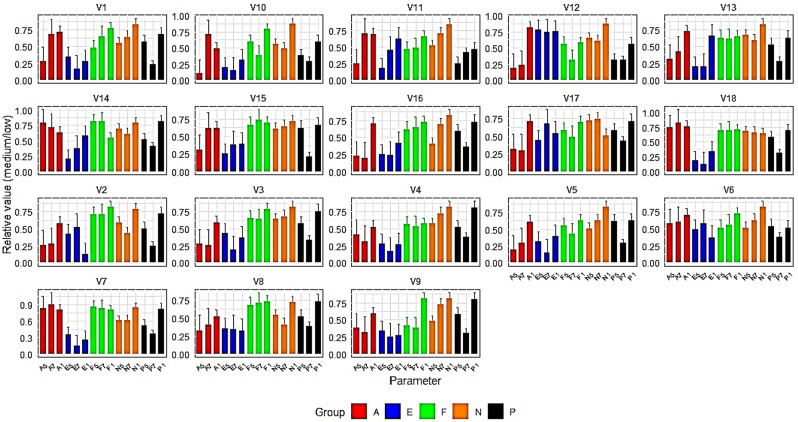
Bar plots illustrating the distribution of relative means (medium/low salinity) within varieties for five photosynthetic parameters: nonphotochemical chlorophyll fluorescence quenching (NPQ), electron transport rate (ETR), maximum potential quantum efficiency of photosystem II (F_V_/F_M_), CO_2_ assimilation rate (A), and photosynthetic performance index (PI_ABS_). The data were collected at three distinct growth stages, namely, stage 1 (leaf 3), stage 5 (boot), and stage 7 (late flowering before silage harvest). The legend key utilizes initials to represent photosynthetic parameter abbreviations, while the digits correspond to the growth stages. The mean values, calculated from six biological replicates, are depicted on the Y-axis. The error bars are the standard deviations

The pre-experiment phenotypic variations observed were more pronounced on the medium-salinity than on the low-salinity platform (Additional File 3). During the experiments, a consistent downward and significant trend in the PI_ABS_ was revealed from stage 1 to stage 7 for all the sorghum varieties. The commonly used stress indicator F_V_/F_M_ exhibited a diverse trend, with fifteen varieties displaying a decrease in expression. This trend was evident for ten varieties for A, fourteen for ETR, and sixteen for NPQ. From leaf 3 to boot, a decrease in the PI_ABS_ was observed in all the varieties except for the local variety 6. Additionally, sixteen varieties exhibited a general decrease in A, with most of these reductions being statistically insignificant. For ETR, half of the varieties demonstrated an increase, while the other half exhibited a decreasing trend with varying levels of significance. Although these decreases were not statistically significant, for NPQ, sixteen varieties exhibited a mixture of trends with varying significance levels. After the plants were transitioned from boot to flowering, twelve varieties displayed an increase in A, and fourteen varieties exhibited an increase in ETR. NPQ showed a mix of both rising and declining trends, the majority of which were not statistically significant, except for variety 16. Conversely, all the varieties experienced a substantial decrease in PI_ABS_, except for the local variety V11, which exhibited a slight increase while V6 showed no change (Fig. 2, Additional File 4).

**Fig. 3:**
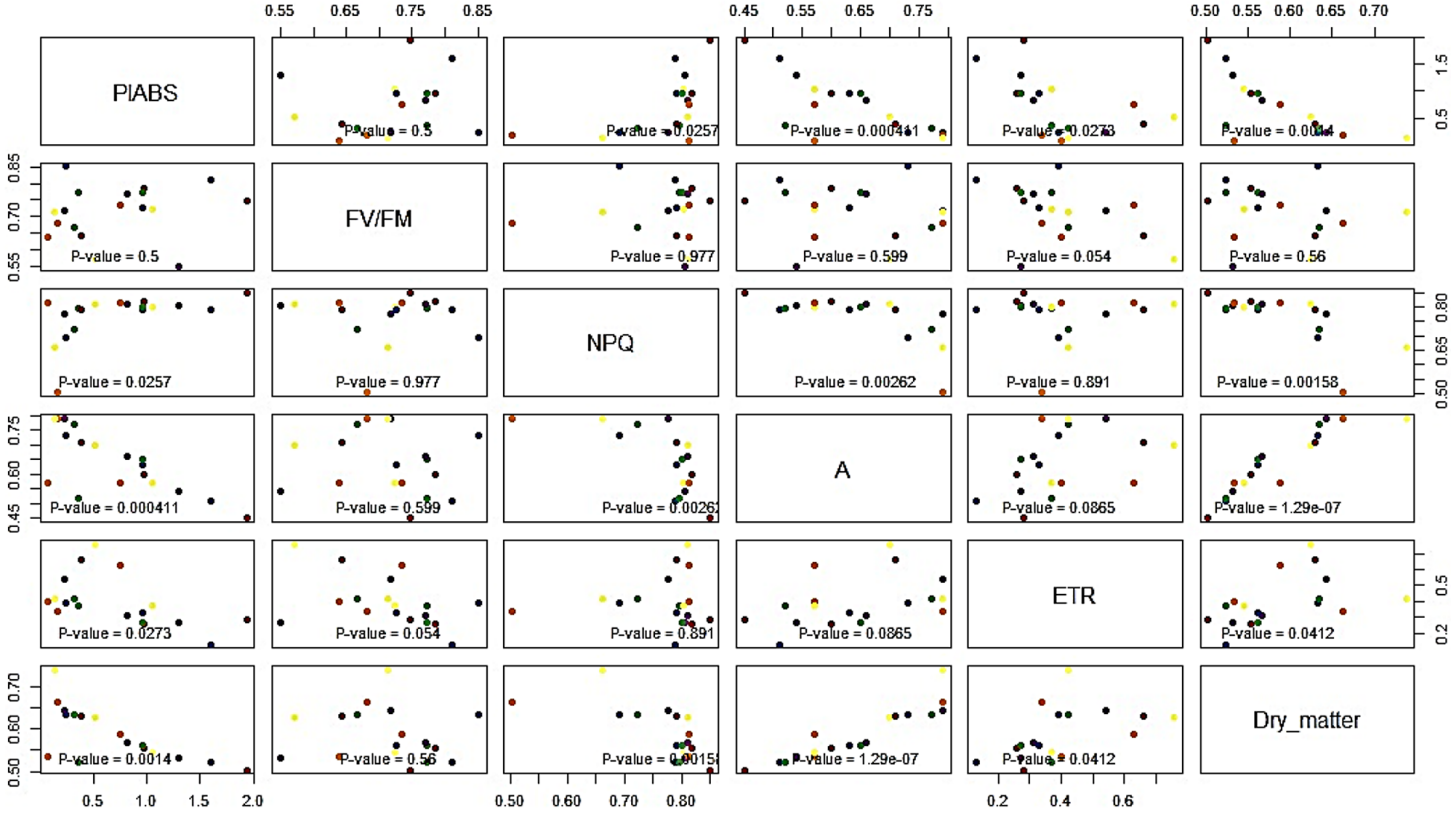
A Scatter plot matrix showing the correlation of relative means (medium/low) at late flowering before silage harvest. The y-axis represents the values of the variables. Each row in the data frame corresponds to a different observation or data point, and the values along the y-axis represent the specific numerical values of each variable for those observations

These findings underscore the high potential of the PI_ABS_ as a parameter for evaluating salt stress sensitivity within the examined sorghum varieties. Further, a correlation analysis revealed significant associations between various photosynthetic parameters and dry matter. Notably, PI_ABS_ exhibits statistically significant correlations with NPQ, A, and dry matter. F_V_/F_M_, on the other hand, does not show significant correlations with other variables. NPQ demonstrates significant relationships with A and dry matter. The A showed significant correlations with PI_ABS_ and dry matter, suggesting a potential link between the efficiency of light absorption and biomass production. Moreover, the NPQ is significantly associated with both A and dry matter. Interestingly, the electron transport rate (ETR) displays a significant correlation with dry matter, while F_V_/F_M_ and ETR exhibited less pronounced associations, their significance in relation to other variables may indicate nuanced interdependencies within the photosynthetic system with PI_ABS_ standing out (Fig. 3).

**Fig. 4:**
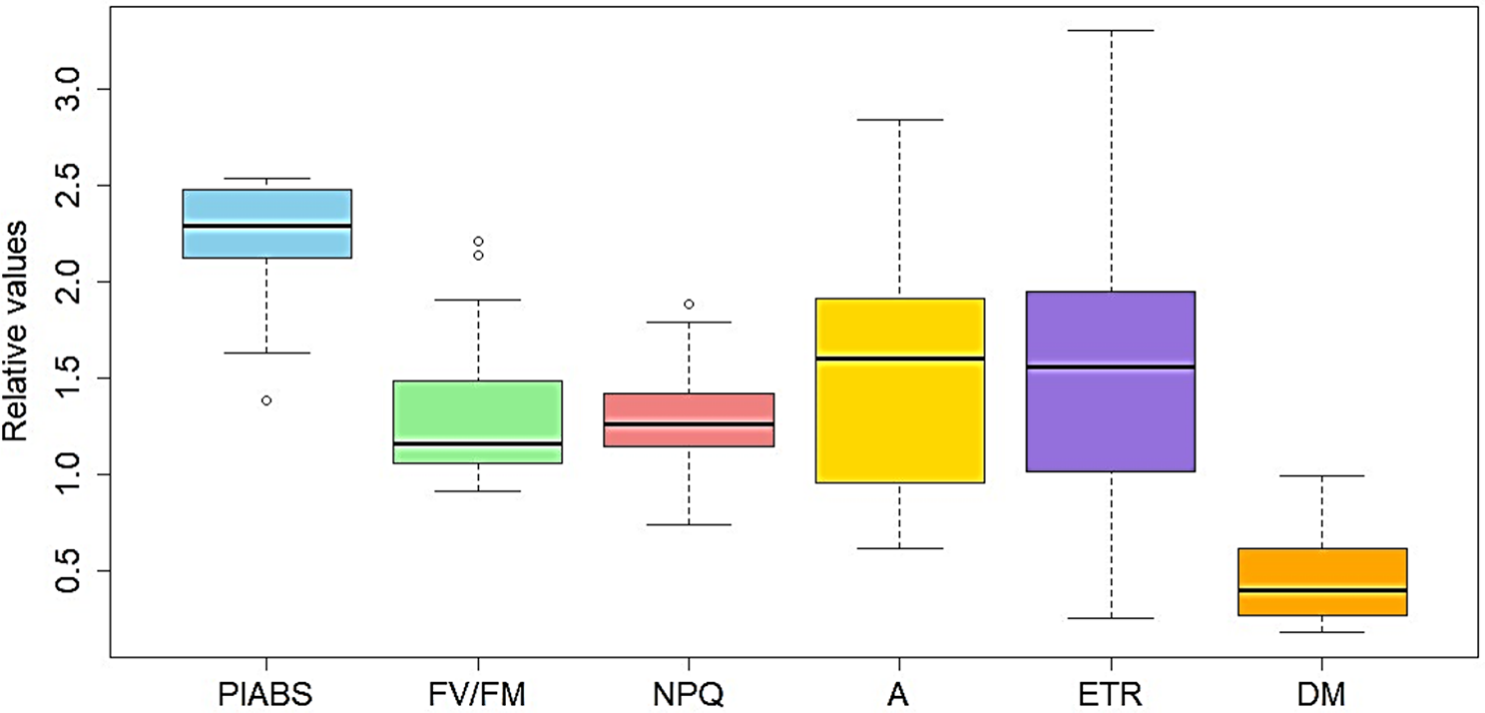
Boxplot showing the relative reduction factors (RFs) (medium/low) calculated by dividing the relative values at leaf 3 by those at flowering, while DM is the relative value after silage harvest in the medium/low salinity

Across the two years of study, analysis of means showed variability in reduction factors among sorghum varieties, highlighting genotype-specific photosynthetic responses to saline conditions. Notably, 83% of the varieties show significant reductions at different stages of growth. The PI_ABS_ demonstrates the highest average reduction factor of 2.3, indicating significant salt stress influence on the sorghum plants’ light absorption efficiency as time progresses. Following closely, parameter A exhibits a substantial impact on electron transport rate. In comparison, both ETR and NPQ reflect moderate reductions, suggesting moderate stress levels or variability across the varieties. Conversely, F_V_/F_M_ shows the lowest average reduction, indicating relatively less impact on photosynthetic efficiency. This trend suggests that the primary photochemical processes and efficiency of energy transfer within photosystem II (PSII) are sensitive to progressive salinity (Fig. 4, Additional File 5).

**Fig. 5:**
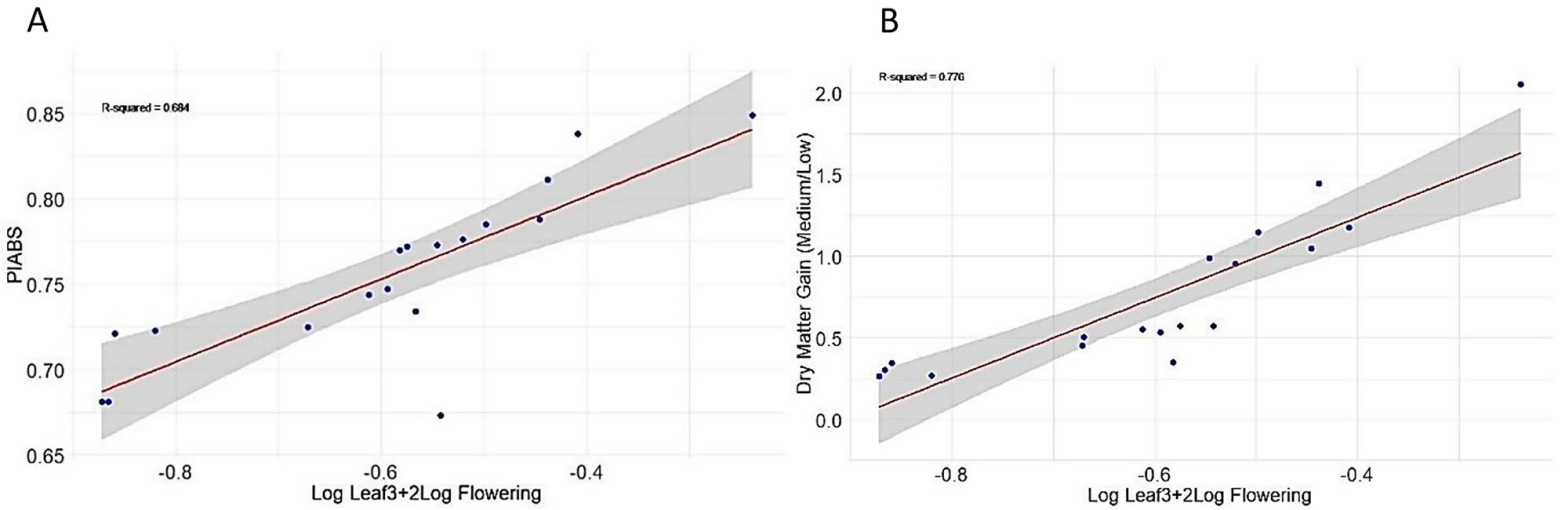
Confidence region plots show the salt performance indices as a function of the photosynthetic performance indices (A) and dry matter yield (B)

As PI_ABS_ serves as a measure of overall photosynthetic performance, its significant reduction across all developmental stages and varieties renders it the most reliable indicator of overall photosynthetic efficiency under salt stress. The PI_ABS_ had the highest significant reduction factor of approximately 2. Therefore, to quantify the SPI, the significant reduction factor “n” was set at 2 in the equation. This approach has proven effective in ranking various soybean and barley genotypes for chilling and drought tolerance, respectively [27, 28]. This implies a double reduction in PI_ABS_ induced by salt stress at flowering compared to leaf 3 across all varieties. As expected, salt-sensitive varieties, which exhibit significant reductions in selected photosynthetic parameters at flowering, will correspondingly exhibit the lowest SPI values. Phenotypically, beyond *n*, we observed that the most salt-sensitive varieties had begun to undergo physiological decline, including withering, chlorosis, and drying, while the tolerant varieties continued to develop. A positive and strong correlation was observed between SPI and relative PI_ABS_, with an R^2^ value of 0.68. Additionally, we investigated SPI concerning relative dry matter gain, represented by the ratio of dry matter mass at medium salinity to that at low salinity. A higher dry matter ratio suggests enhanced performance under saline conditions. Notably, there existed a positive correlation between dry matter gain and SPI, with an R^2^ value of 0.77. (Fig. 5).

### Effect of rubisco genes on the PI_ABS_

The *Rubisco* gene family plays a pivotal role in the initial stages of carbon fixation during photosynthesis, exhibiting high functional conservation [40]. We aimed to explore whether its transcriptional regulation could pleiotropically influence the PI_ABS_, highlighting an interconnection of carbon fixation and light energy utilization in photosynthesis. We specifically targeted variety 3 for transformation due to its comparatively lower photosynthetic performance in terms of CO_2_ assimilation and PI_ABS_ and the observed transcriptional downregulation of Rubisco 8, a gene that showed upregulation in the highest-performing variety. By augmenting the expression of the Rubisco gene in this variety, we anticipated discernible alterations in transcriptional regulation, as well as potential phenotypic variations in the sensitive OJIP with influence on PI_ABS_, and potential A, in comparison to the agriculturally superior variety. The PI_ABS_ is a highly quantitative trait presumably regulated by multiple genes. Due to the strong positive correlation between PI_ABS_ and A and between A and dry matter, we assessed whether the transcriptional regulation of A has an influence on the PI_ABS_. CO_2_ assimilation is regulated by rubisco genes, but little research has been conducted on the genetic control of A in sorghum. We therefore used bioinformatics to search for conserved regions of Rubisco genes and validated the results via real-time qPCR. After filtering out redundant sequences, a total of eight rubisco large subunit genes (rbcL) were identified and renamed *SbrbcL1* to *SbrbcL8*. The physicochemical property analysis showed that the molecular weight ranged from 19058.9 to 46472.2 kDa. The pI values were greater than 6, indicating that all the rubisco proteins were basic proteins. The genes were distributed in chloroplasts, the cell membrane, and the endoplasmic reticulum (ER) (Table 2, Additional File 6).

**Table 2:** Rubisco gene characteristics

Designation	Gene ID	Chromosome	Start: end	Amino acid no.	Instability Index	Aliphatic index	GRAVY	pI	Mw	Location
*SbrbcL1*	SORBI_3005G042000	5	3876057:3877378	169	46.75	71.6	-0.244	8.77	19058.87	Chloroplast
*SbrbcL2*	SORBI_3001G283700	1	55609081:55626018	448	52.82	86.67	-0.266	6.48	49789.79	Chloroplast
*SbrbcL3*	SORBI_3001G206000	1	18808475:18811149	305	52.43	98.46	0.299	10.8	32970.79	ER
*SbrbcL4*	SORBI_3006G221500	6	56819175:56821962	289	39.51	107.75	0.227	10.2	31462.90	ER
*SbrbcL5*	SORBI_3003G145900	3	15004204:15009068	377	48.39	89.1	-0.098	8.59	41386.42	ER
*SbrbcL6*	SORBI_3009G188900	9	54103037:54106174	406	47.14	93.82	0.08	8.4	46472.22	ER
*SbrbcL7*	SORBI_3009G108500	9	43527964:43532814	396	59.13	89.67	-0.043	7	43581.97	Chloroplast
*SbrbcL8*	SORBI_3003G317500	3	64515754:64520205	399	44.08	98.62	-0.002	8.92	45974.68	Cell membrane

**Fig. 6:**
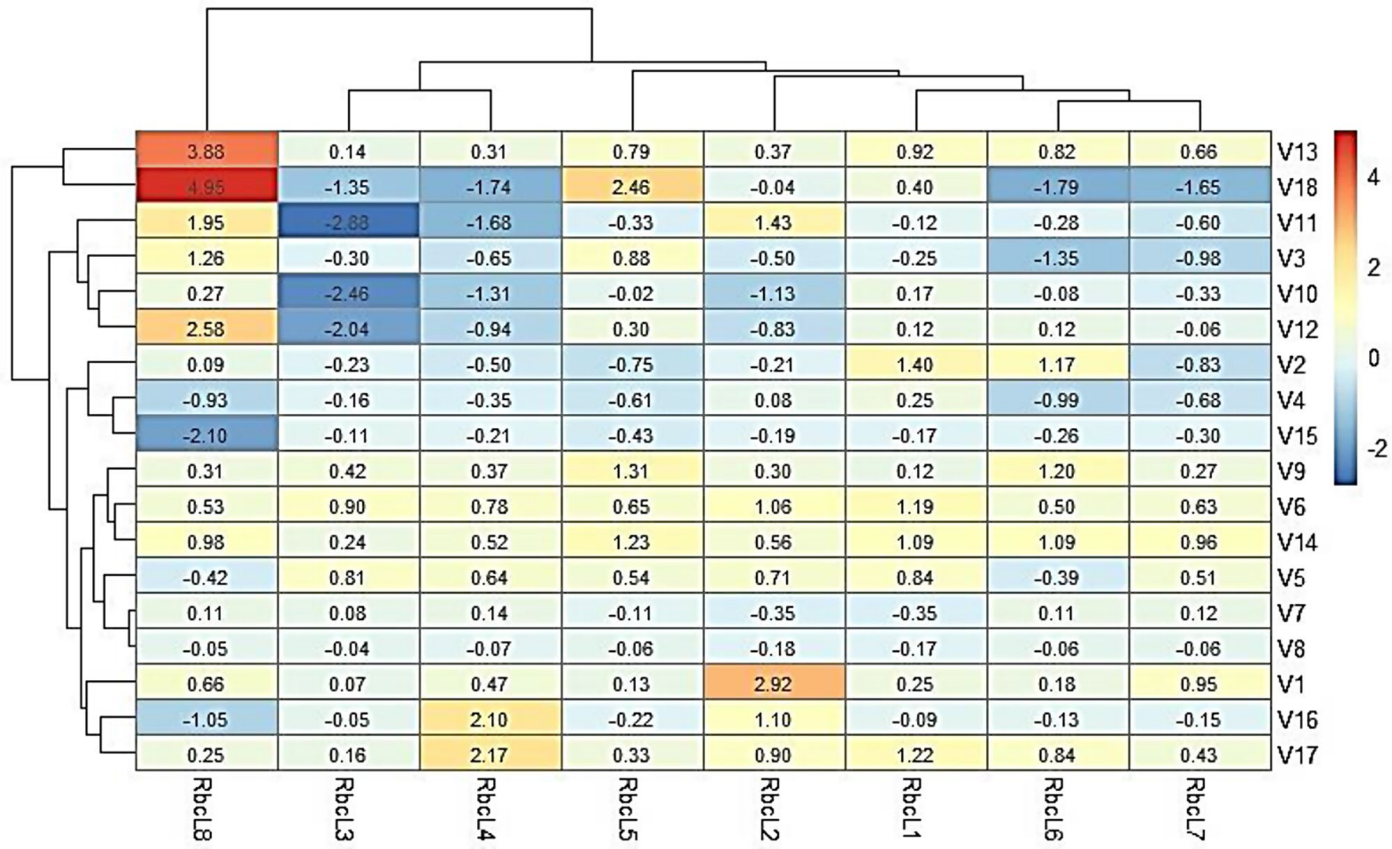
A cluster heatmap of the relative expression of the rubisco genes detected across the varieties under medium salt stress generated via real-time qPCR. Each row represents a variety, while columns represent individual genes. The intensity of the colors indicates the regulation level (e.g., red indicates upregulation, and blue indicates downregulation). The values are relative to low-salinity conditions calculated via the 2^−ΔΔ*CT*​^ method using the *EF1* gene as the internal control

Real-time qPCR analysis using gene-specific primers (Additional File 7) revealed that all the genes were present and highly expressed on the leaves (Fig. 6). Under salt treatment, there was remarkable variation in the expression level of the rubisco genes, with V13 and V18 exhibiting the highest and second highest upregulation of *SbrbcL8* and *SbrbcL5*, respectively, while V11 and V10 exhibited the highest downregulation of *SbrbcL3*. There are two notable expression clusters. The first cluster consists of V1, V2, V4, V5, V6, V7, V8, V9, and V15. There are also two hybrids, V16 and V17, and an inbred line, V14. In this cluster, *SbrbcL2* was highly upregulated in the local variety V1, while *SbrbcL4* was highly upregulated in the hybrids V16 and V17. Additionally, within this cluster, *SbrbcL1* was upregulated in the local variety V2. The second cluster consists of V3, V10, V11, V12, V13, and V18. This cluster included the most upregulated genes, with *SbrbcL8* being upregulated in the V11, V12, and V13 varieties, as well as in the hybrid V18 variety (Fig. 6).

### Overexpression of *SbrbcL8* is associated with altered growth and increased PI_ABS_

**Fig. 7:**
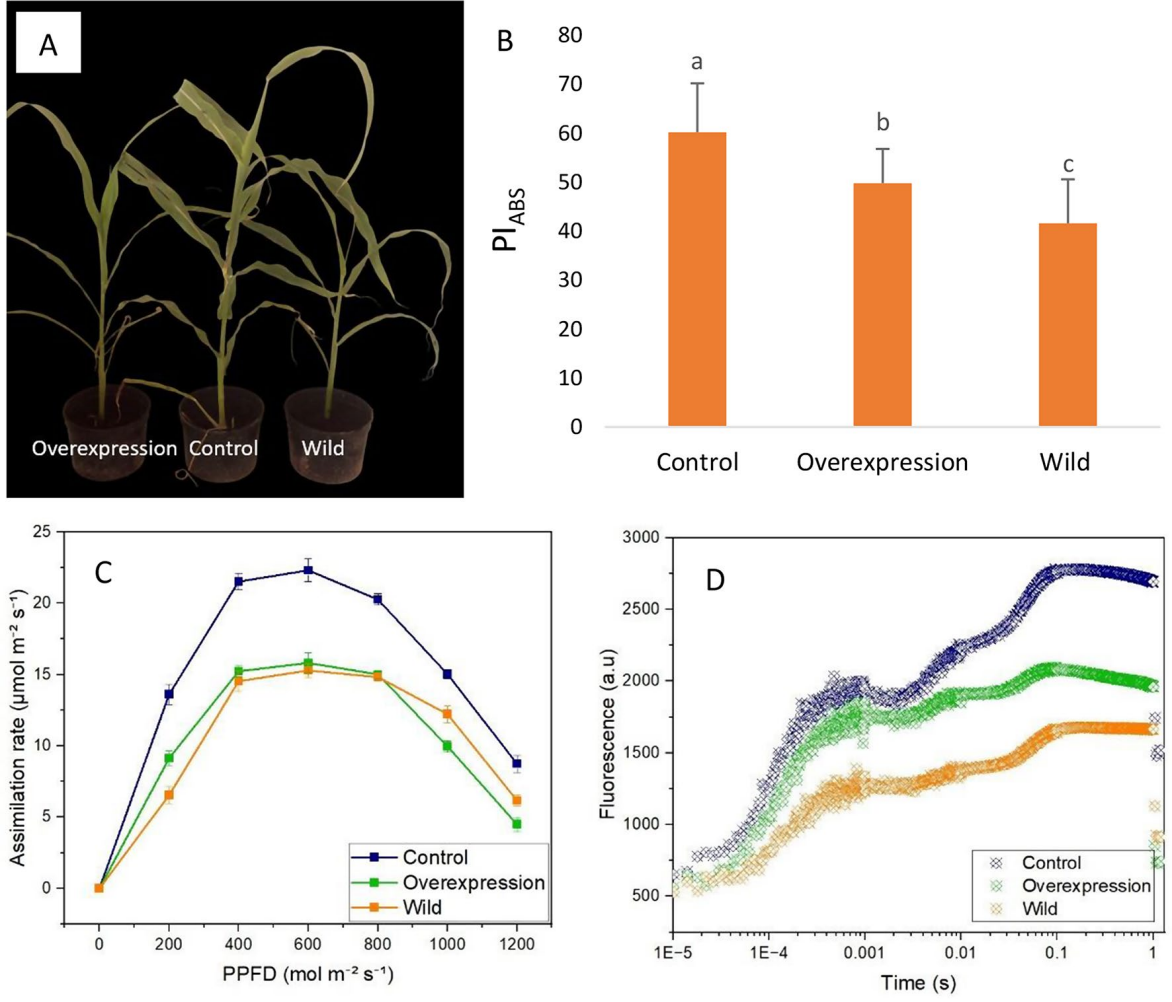
Graphical representation of (A) morphology, (B) PI_ABS_ variation, (C) CO_2_ assimilation rate, and (D) chlorophyll a fluorescence transient curve for the control (0 dS/m NaCl), wild-type (8 dS/m NaCl), and overexpressed (*GUS::SbrbcL8*, 8 dS/m NaCl) treatments. The values for each point were means ± SD (*n* = 6). Significant differences are indicated by different letters (ANOVA; P values ≤ 0.05)

Understanding the rationale behind gene overexpression compared to the wild type, particularly under basal and stress conditions, is pivotal in elucidating its biological significance and practical applications. The growth patterns of the overexpressed, wild type, and control plants exhibited noticeable differences under salt stress conditions. Plant growth was slowed/arrested in wild type after salt treatment, while the overexpressed and control plants exhibited normal growth (Fig. 7A). Additionally, the homologous overexpression of *SbrbcL8* in the local variety V3 resulted in altered chlorophyll fluorescence and an increase in PI_ABS_. In brief, all the plant samples displayed a polyphasic increase in Chl fluorescence after dark adaptation within the first second of illumination with high-intensity light. The increase was greater in the overexpressed plants than in the wild type but lower than that in the control (Fig. 7). Furthermore, we measured the photosynthesis rate by elevating CO_2_ levels. There was a steep and linear increase in net carbon assimilation as the internal CO_2_ concentration increased. However, the values were insignificant for overexpressed and wild type (Fig. 7B-D). This observation suggested that *SbrbcL8* overexpression results in an increase in the PI_ABS_, which not only acts as the driving force of photosynthesis, as demonstrated in the present study but also enhances salt performance and dry matter accumulation.

## Discussion

Understanding plant genetic diversity is crucial for enhancing crop improvement programs [41]. Also, the major challenge is identifying desirable genotype in addition to sufficient genetic material. However, obtaining sufficient and suitable genetic material poses a significant challenge, resulting in difficulties in developing the best-performing varieties [42]. In this study, 18 sorghum varieties demonstrated diverse performances under low and medium salinity levels. This result provides an opportunity to explore reliable photosynthetic markers for salt tolerance and anticipate measures for genetic improvement. The slow kinetics of chlorophyll fluorescence are highly sensitive to environmental changes [43]. Many studies on chlorophyll fluorescence have accurately predicted crop yield under different environmental conditions [44, 45]. The F_V_/F_M_ indicates the dark-adapted leaf prephotosynthetic fluorescent state and has implications for pathways related to crop yield and stress tolerance [46].

In the present study, salinity progression had a variable negative effect on the F_V_/F_M_ values of the varieties. The maintenance of high relative F_V_/F_M_ values at leaf 3 compared to the boot stage indicated that the impact of salt stress was lowest at this growth stage. As growth progressed, the F_V_/F_M_ values decreased across the accessions, suggesting photoinhibition from abiotic stress [47]. The NPQ is a molecular adaptation that represents the rapid reaction of the photosynthetic membrane to excess light to overcome or prevent photoinhibition [48]. NPQ is directly or indirectly connected to processes such as light capture, energy transfer, electron transport, proton relocation, ATPase activity, and carbon incorporation, influencing overall plant photosynthesis, biomass, and yield [49]. In the present study, the weak correlation between NPQ and F_V_/F_M_ suggested that the photoprotective processes involved in NPQ, which prevent damage to the photosynthetic apparatus under excessive light conditions, may not be directly linked to the variation in the maximum photochemical efficiency of PSII at silage maturity. Furthermore, the low correlation between NPQ and ETR can be attributed to the fact that dark photosynthesis typically involves less energy flux than light-dependent photosynthesis [50].

In the absence of light, the electron transport chain may be less active, leading to a reduced demand for NPQ mechanisms to dissipate excess energy [51]. All the parameters except for F_V_/F_M_ were positively and significantly correlated with yield. This indicates that the efficiency of photosynthetic light energy conversion, as measured previously, may not be a strong predictor of the overall productivity or yield of a crop. Taken together, these findings suggest that under salt stress, sorghum may prioritize protective mechanisms such as NPQ over maximizing photosynthetic efficiency. These findings support the findings of Van Heerden and Krüger [52], who further argued that the reliance of F_V_/F_M_ on F_0_ also made them insensitive for assessing mildly stressed plants.

In mildly stressed environments, the PI_ABS_ has proven to be a highly appropriate and sensitive metric for investigating overall plant photosynthetic capacity [53]. PI_ABS_ integrates three independent parameters, namely, the density of fully active reaction centers, the efficiency of electron transport, and the probability of an absorbed photon being trapped by the reaction center [54]. This parameter reflects the functionality of both PSI and PSII, providing quantitative information on the current state of plant performance under stress conditions [55]. In the present study, the PI_ABS_ exhibited a linear and significant decline from leaf 3 to flowering, which was positively correlated with NPQ and dry matter yield. The highly significant positive correlation between PI_ABS_ and A indicates the presence of an efficient electron transport chain, which supports the generation of ATP and NADPH required for CO_2_ fixation during the Calvin cycle, as indicated by the greater A and ETR. Thus, the PI_ABS_ in this study was a reliable salt performance indicator in terms of salt stress tolerance and yield.

As a quantitative trait, PI_ABS_ is likely regulated by numerous genes within the photosynthetic pathway, encompassing processes from initial light capture in light-harvesting complexes (LHC) and photochemical reactions to subsequent CO_2_ assimilation and growth [56]. Considering the direct link between CO_2_ assimilation and biomass accumulation, investigating the potential transcriptional regulation of CO_2_ fixation in sorghum is imperative for understanding PI_ABS_ behavior and dry matter. While the rubisco gene family is highly conserved during the CO_2_ assimilation process [57], no studies have been performed on this gene family in sorghum. We therefore used bioinformatics to discover rubisco genes in sorghum through multiple sequence alignment and validated the results via qPCR. Among the genes, *SbrbcL8* is highly expressed in commercial variety 18 and was used to transform local variety 3. The *SbrbcL8*-overexpressing regime exhibited enhanced PI_ABS_ activity and altered OJIP behavior. There was also an increase in A, but it was not significant. These collective observations suggest that the molecular components of chloroplast photoprotection and CO_2_ assimilation are closely related to yield in sorghum. This study establishes, for the first time, a genetic link between PI_ABS_ and A, a critical stage in sorghum improvement for photosynthetic performance.

## Conclusion

In conclusion, this comprehensive study on sorghum genetic diversity, salt tolerance, and photosynthetic performance provides valuable insights into the intricate relationships among various parameters. The analysis of molecular markers revealed substantial genetic diversity among local landraces with higher allelic richness, gene diversity, and observed heterozygosity. Population structure analysis identified two distinct subpopulations with significant admixture, emphasizing the need for considering genetic diversity in breeding programs. The slow kinetics of chlorophyll a fluorescence and CO_2_ assimilation parameters were examined across different developmental stages, revealing variations in response to salt stress among sorghum varieties. Notably, the PI_ABS_ emerged as a robust salt performance indicator. The correlation analysis further highlighted the connections between PI_ABS_ and other photosynthetic parameters, emphasizing its role in assessing salt stress sensitivity and biomass accumulation. The genetic control of PI_ABS_ was explored through the investigation of rubisco genes, revealing distinct expression patterns among different sorghum varieties under salt treatment. The overexpression of *SbrbcL8* in a local variety resulted in altered chlorophyll fluorescence, increased PI_ABS_, and slight changes in CO_2_ assimilation rates. This suggests a direct genetic link between rubisco genes, PI_ABS_, and photosynthetic efficiency in sorghum, providing novel insights for future breeding strategies. The findings pave the way for targeted breeding efforts like CRISPR-mediated genome editing to enhance salt tolerance and optimize photosynthetic performance in sorghum, a crucial step towards sustainable crop improvement in the face of increasing salinity problem.

### Electronic supplementary material

Below is the link to the electronic supplementary material.

Supplementary Material 1

## Data Availability

All the data are included in the manuscript and its supplementary information files.
